# Supplementary material for: A qualitative evaluation of treatment fidelity alongside a pilot trial of a novel therapy for pediatric Inflammatory Bowel Disease
Source: PLoS One. 2024 Jul 30;19(7):e0292709. doi: 10.1371/journal.pone.0292709 (PMC11288461; doi:10.1371/journal.pone.0292709)
Supplement: S1 File — (DOCX) [file pone.0292709.s001.docx]

**Caregiver Interview Guide**

**Introduction to the study**

Thank you for taking the time to speak with me today. I’m a research coordinator at the Ottawa Hospital Research Institute working with Dr. Presseau, a health psychologist and scientist who also works at the Ottawa Hospital Research Institute. We’ve been working with Dr. Mack and the staff at the CHEO IBD Centre on a study that is looking at how people manage when they’re asked to do several things as part of a research study.

Specifically, we’re interested in your experiences participating in the resistant starches study. We would like to know more about how you managed with helping your child do things like take their IBD medications, keep up with taking the resistant starches, collecting stool samples, and keeping a symptom diary on top of everything else you do as a caregiver. We know it can be challenging so we’d like to know more about how it went for you. As part of this interview study, we are speaking with people who have participated in the resistant starches pilot trial, like yourself, to get a sense of what the experience was like.

We’re hoping to learn from you about what helped you and what was challenging about participating in the trial so that we can better prepare for future trials that may be done at other IBD centres. In other words, our goal is to learn more about the ways we can better support families who choose to participate in these types of trials.

Did you have any questions or concerns about what we just covered?

**Participant rights and consent**

Okay great, now I just want to emphasize some key points that were explained in the consent form. First, please know that your participation is totally voluntary. That means that at any point during the interview you can decide you no longer want to participate or that you do not want to answer any questions. I want you to also know that whether you choose to participate or not will not affect the care you receive at the CHEO IBD Centre.

The interview will take about 30-60 minutes, depending on how much you would like to share.

You should know I will record our interview today to make sure we get an accurate account of what you share and so that we can type it up later. Once we have the interview typed up we will take out any mentions of places, names or unique details that may make your identity known to others.

Your privacy is important to us so we will do everything in our power to keep our interview confidential. That means that only the members of the research team who are based at the OHRI and a transcriptionist who has signed a confidentiality agreement will be able to listen to your interview recording or see your transcript.

Members of the research team who are based part of the CHEO IBD clinic won’t have access to the interview audio or transcript, but will see summaries of the results including quotes from your interview. For example, during research meetings I may share that half of the people I’ve spoken to so far think blue is a nice colour. Or I may say, two people have found playing soccer challenging. When we present the results more formally (e.g., academic journals) statements like these will include quotes from interviews.

So, to further protect your identity, we will ask that you provide us with a pretend or fake name that we can use with your quotes. What fake name would you like to use?

Fake name: _________________________

What pronoun (e.g., he, she, they) would you like to use with that name?

Pronoun: _____________________

And as a thank you for participating, you and your child will get a $25 gift card (one per family).

You may choose from: ____ Tim Hortons ____Chapters _____ Amazon

Any questions about anything we’ve covered so far?

Okay great. Please know **there are no right or wrong answers**, I’m really just interested in hearing about what you think. Also, please keep in mind that I am **not a clinician,** my background is in social psychology, so I may ask for clarifications from time to time.

Any questions before we start?

**[begin recording**]

May I get your **consent** to proceed with the interview **on the record**?

**Part 1 – Introduction to Trial**

I’d love to know more about what participating in the resistant starches study has been like for you.

1. Thinking back, how did you first hear about the resistant starches study?
   1. How was it introduced to you? By whom?
2. What made you decide to participate in the resistant starches pilot trial?
3. Can you tell me a bit about, what’s it been like to be part of the study for the past few months?
4. What were you and your child asked to do as part of the resistant starches study?
5. What instructions were you and your child given (i.e., how did you know what you were supposed to do)?
   1. [use visual aids – e.g., can you tell me a bit about how you used this brochure?]
   2. Was there anything that you were taught to do so you could be part of the RS study?
6. What resources or skills training did you receive, if any, to help you be part of the resistant starches study?

**Part 2 –** **Experiences with Trial Activities**

Now I’d like to know a bit more about your experiences with some of the specific activities you and your child were asked to do as part of the trial. I’m interested in hearing about what it was like helping your child take their prescribed medications, take the daily resistant starch dose, collect stool samples, and keep track of their doses and symptoms using the study brochure.

1. What was your role in helping your child do each of these four activities?
   1. How did you help/support your child, if at all?
   2. What was your child responsible for doing?
   3. Who else was involved in helping your child complete these four tasks?
2. Tell me about your experiences supporting your child to take their prescribed medications.
   1. What were the instructions you and your child received for taking the medications?
      1. Tell me about the medication regimen (e.g., different meds, doses, timing).
   2. What went well?
   3. What did not go so well?
   4. Tell me about a time when they did not take their prescribed medications.
      1. What happened? What got in the way?
   5. What was the most difficult part about helping your child take their medications?
3. Tell me about your experiences helping your child with taking the resistant starches on a daily basis.
   1. What were the instructions you were given for helping your child take the resistant starches?
   2. What went well?
   3. What did not go so well?
   4. Tell me about a time when your child did not take the resistant starches.
      1. What happened? What got in the way?
   5. What was the most difficult part about helping your child take the resistant starches?
4. Tell me about your experiences with collecting the stool samples.
   1. What instructions were given to you for helping your child collect stool samples?
   2. What went well?
   3. What did not go so well?
   4. Tell me about a time when you/your child were supposed to collect a sample and did not collect one.
      1. What happened? What got in the way?
   5. What was the most difficult part about helping your child collect the stool samples?
5. What about tracking doses and symptoms using the brochure? Tell me about your experiences helping your child use the tracking sheets from the brochure.
   1. What instructions were you given for helping your child keep a symptom and dose diary?
   2. What went well?
   3. What did not go so well?
   4. Tell me about at time when entries were not recorded.
      1. What happened? What got in the way?
   5. What was the most difficult part about helping your child keep a symptom and dose diary?
6. Which of the four activities (meds, powder, poop, diary) was the easiest/hardest to do? What made it easy/difficult?

**Part 3 – Managing Competing Demands**

Now I’m interested in hearing more about how you managed to help your child with these activities along with everything else you do as a caregiver.

1. What was it like for you to help your child stay on top of the four activities we’ve discussed?
2. What was it like helping your child make the four activities part of your routine?
   1. What strategies or tools did you use?
   2. What was challenging? What was easy?
3. Thinking back over the past few months, what else was going on in your life at the time that made it challenging to do the activities we’ve been talking about?
   1. How did you manage to continue helping your child with the study tasks while dealing with all the demands on your time?
   2. How did these competing demands affect your ability to support your child with the activities we’ve been talking about?
   3. What have you had to change to make room for these activities (medication, starches, diary) into your daily life?
   4. What has been helpful/rewarding?
4. [If withdrew from trial] Tell me more about what was happening when you withdrew from the trial.
   1. What led you to withdraw?

**Part 4 – Reflecting on Trial Experiences**

1. Thinking back over the past few months, what would have made any of the four activities we’ve talked about easier to do?
   1. What material resources would have been helpful?
   2. What information would you have liked to have had?
   3. What kind of social supports would have been helpful?
2. How would you describe your overall experience helping your child participate in the study so far?
   1. What has gone well for you?
   2. What has gone not so well/could be better?
3. What advice would you give to other people who are considering participating in a trial like this?

Is there anything else you’d like to share with me today?

Before we say goodbye, I just wanted to check in with you to see how you’re feeling.

If you feel like you would like to speak to someone about how you are feeling, I encourage you to contact your mental health provider, if you have one. You may also contact the Distress Centre of Ottawa. They can help you process your feelings and connect you to additional resources if you need them.

If you are in distress, you can reach the Distress Centre of Ottawa at: 613-238-3311

If you are experiencing a crisis, you may call them at: 613-722-6914 or 1-866-996-0991

You may also TEXT them at 343-306-5550 between 10am-11pm.

We also have a list of IBD and mental health related resources that may be useful to you. I will send those along by email.

Thank you so much for taking the time to speak with me today. What you shared with me today has been incredibly helpful.

**Youth Interview Guide**

**Introduction to the study**

Thank you for taking the time to speak with me today. I’m a research coordinator working with Dr. Presseau, a scientist at the Ottawa Hospital Research Institute. We’ve been working with Dr. Mack and the staff at the CHEO IBD Clinic on a study that is looking at how people manage when they’re asked to do many things as part of a research study.

Specifically, we’re interested in hearing what it was like for you to participate in the resistant starches study. We would like to know more about what it was like taking your medications, taking the resistant starch powder, collecting stool samples, and keeping track of your doses and symptoms using the study brochure. We know it can be challenging to do all those things on top of everything else you do so we’d like to know more about how it went for you. As part of this study, we are speaking with people who have participated in the resistant starches study, like yourself, to get a sense of what the experience was like.

We’re hoping to learn from you about what helped you and what was hard about participating in the trial so that we can better prepare for other research studies that may be done at other IBD centres. In other words, we want to know what we can do to make it easier to participate in research studies like the resistant starches trial.

Did you have any questions about anything I’ve said so far?

**Participant rights and consent**

Okay great, now I just want to go over some key points that were talked about in the consent form. First, please know that your participation is totally voluntary. That means that at any point during the interview you can decide you no longer want to participate or that you do not want to answer any questions. I want you to also know that whether you choose to participate or not will not affect the medical care you get at the CHEO IBD clinic.

The interview will take about 30-60 minutes, depending on how much you would like to share.

You should know I will record our interview today so that we can type it up later. Once we have the interview typed up we will take out any mentions of places, names or specific details that may make your identity known to others.

Your privacy is important to us so we will do everything in our power to keep our interview confidential. That means that only the members of the research team who are based at the OHRI and a transcriptionist who has signed a confidentiality agreement will be able to listen to your interview recording or see your transcript.

People who are part of the research team who work at the CHEO IBD clinic won’t be able to listen to your interview recording or see your transcript, but they will see summaries of the results, including quotes from your interview. For example, during research meetings I may share that half of the people I’ve spoken to so far think blue is a nice colour. Or I may say, two people have found playing soccer challenging. When we present the results more formally (e.g., academic journals) statements like these will include quotes from interviews.

So, to better protect your identity, we will ask that you provide us with a fake name that we can use with your quotes. What fake name would you like to use?

Fake name: _________________________

What pronoun (e.g., he, she, they) would you like to use with that name?

Pronoun: ___________________

And, as a thank you for participating, you and your caregiver will get a $25 gift card (one per family).

You may choose from: ____ Tim Hortons ____Chapters _____ Amazon

Any questions about anything we’ve covered so far?

Okay great. Please know **there are no right or wrong answers**, I’m really just interested in hearing about what you think. Also, please keep in mind that I am **not a clinician,** so I may have to ask you to explain things to me that I am not familiar with.

Any questions before we start?

**[begin recording**]

May I get your **consent** to proceed with the interview **on the record**?

**Part 1 – Background**

So, I hear you have been participating in the resistant starches study.

1. How did you first hear about the resistant starches study?
   1. What did they tell you about it?
2. What made you decide to participate in the resistant starches study?
3. Can you tell me a bit about, what’s it been like to be part of the study for the past few months?
4. What were you asked to do as part of the resistant starches study?
5. What instructions were you given (i.e., how did you know what you were supposed to do)?
   1. [use visual aids – e.g., e.g., can you tell me a bit about how you used this brochure?]
   2. Was there anything that you were taught to do so you could be part of the RS study?

**Part 2 – Trial Activities and managing competing demands**

So, I’m interested in hearing more about what it was like to take your IBD medications, take the resistant starches powder, collect your stool samples, and use the tracking pages in the brochure.

1. Thinking back over the last 6 months, what has it been like taking your IBD meds/resistant starches/collecting stool samples/keeping diary?
   1. What went well?
   2. What did not go so well?
   3. How much work was it for you to do these activities?
2. How did you make these activities part of your daily routine?
   1. Tell me about it, what was your routine?
   2. How much was work was it for you to make these activities part of your routine?
   3. What tricks and strategies did you use to stay on top of things?
      1. Probe for reminders, tools, social support, habit
3. Which of these activities (meds, powder, poop, diary) did you find the hardest to do? What made it hard?
   1. What happened when you ran into problems?
   2. Was there anyone who helped you when you ran into problems with ____ [meds, RS, poop, diary]
4. Which was the easiest of the four activities (meds, powder, poop, diary) to do? What made it easy?

We know it can be hard to keep up with everything all of the time. I would like to better understand what happens when people are not able to do things like take medications or collect stool samples so we can plan better ways of supporting future study participants.

1. Can you tell me about a time when you were not able to do one of the activities (you pick which)?
   1. What happened? What got in the way?
   2. What happened as a result of missing the activity (e.g., medication dose)?

Now I’m interested in hearing more about how you managed to do these activities along with everything else going on in your life.

1. Sometimes, life gets pretty busy. I’m interested in knowing more about what those moments were like for you.
   1. What made your life so busy?
   2. What was most important to you during that time?
   3. How did you manage (or not) to continue doing the things we’ve been talking about?
2. How have school/work/friends/extracurricular activities affected your ability to do the activities we’ve been talking about (taking medications, taking their resistant starches, collecting stool samples, keeping a symptom/dose diary)?
3. [If withdrew from trial] Tell me more about what was happening when you withdrew from the trial.
   1. What led you to withdraw?

**Part 3 – Reflecting on Trial Experiences**

1. Thinking back over the past few months, what would have made any of the four activities we’ve talked about easier to do?
   1. What material resources would have been helpful?
   2. What information would you have liked to have had?
   3. What kind of social supports would have been helpful?
2. How would you describe your overall experience participating in the study so far?
   1. What has gone well for you?
   2. What has gone not so well/could be better?
3. What tips or advice would you give to other young people who are considering participating in a study like this?

Is there anything else you’d like to share with me today?

Before we say goodbye, I just wanted to check in with you to see how you’re feeling.

If you feel like you would like to speak with someone about your ongoing experiences living with IBD, I encourage you to get in touch with your IBD social worker ___________.

If you feel like you would like to speak to someone sooner about how you are feeling, I encourage you to contact your mental health provider, if you have one, or the Distress Centre of Ottawa. They can help you process your feelings and connect you to additional resources if you need them.

If you are in distress, you can reach the Distress Centre of Ottawa at: 613-238-3311

If you are experiencing a crisis, you may call them at: 613-722-6914 or 1-866-996-0991

You may also TEXT them at 343-306-5550 between 10am-11pm.

We also have a list of IBD and mental health related resources that may be useful to you. I will send those along by email.

Thank you so much for taking the time to speak with me today, those were all my questions. What you shared with me today has been incredibly helpful.

**Child Interview Guide**

**Introduction to the study**

Thank you for taking the time to talk to me today. I work at the Ottawa Hospital with a scientist, Dr. Presseau. We’ve been working with Dr. Mack and the people at the CHEO IBD Clinic to look at what kids like you think about being in the resistant starches study. We would like to know more about what it was like to take your medications, to eat the resistant starches powder, to collect your poop in the containers you were given, and to keep track of your symptoms, or what your body was feeling, in a diary. We know it can be hard to do all those things we’d like to know more about how it was for you. We’re hoping to learn from you about what helped you and what was hard about being in the study so that we can make it even easier for other kids to participate in studies like this.

Did you have any questions about anything I’ve said so far?

**Participant rights and consent**

Okay great, now I just want to go over some important points. First, whether you want to talk to me and do an interview is totally up to you! You don’t have to if you don’t want to, no one will be mad at you or sad if you decide you don’t want to do the interview. You can also decide you don’t want to do the interview anymore at any time, even after we’ve started, you just let me know. You also don’t have to answer questions you don’t want to answer.

The interview will take about 30 minutes but we can make it shorter or longer, up to you.

So, we want to keep your name secret so that it’s hard for people to know what you said. What fake name would you like to use?

Fake name: _________________________

Would you like [fake name] to be called a “he, she, they” or something else?

Pronoun: ____________________

Any questions about anything we’ve covered so far?

Okay great. Please know **there are no right or wrong answers**, I’m really just interested in learning from you.

Any questions before we start?

**[begin recording**]

Do you agree to participate in this interview?

1. So, I heard you have been taking part in a study about resistant starches. Can you tell me about that?
2. What were you asked to do as part of that study?

I’m interested in hearing more about what it was like to take medicine, eat the powder that comes in packets, collect your poop in containers, and keep track of how your body was feeling (symptoms) in a diary.

1. Which of those did you have to do?
   1. Did anyone help you do any of those activities?
2. What was it like to take the medicine your stomach doctor gave you?
   1. How easy or hard was it to take your stomach medicine everyday?
      1. What was easy about taking your medicine?
      2. What was hard about taking your medicine?
   2. Tell me about a time when you did not take your medicine.
      1. What happened?
      2. What got in the way?
3. What was it like to eat the powder that comes in packets?
   1. How easy or hard was it to eat the powder?
      1. What was easy about eating the powder?
      2. What was hard about eating the powder?
   2. Tell me about a time when you did not eat the powder.
      1. What happened?
      2. What got in the way?
4. What was it like to put your poop in the containers you were given? [show visual]
   1. How easy or hard was it to collect poop in jars?
      1. What was it easy about collecting poop?
      2. What was hard about collecting poop?
   2. Tell me about a time when you were going to collect poop in jars but you did not.
      1. What happened?
      2. What got in the way?
5. What was it like to use the study brochure? [show visual]
   1. What was it like to use page 14 [show visual] to keep track of how your body was feeling?
   2. What was it like to use pages 12 and 13 to keep track of when you ate the powder?
   3. How easy or difficult was it to use these pages in the brochure?
      1. What was the hardest part about using the brochure?
      2. What was the easiest part?
   4. Tell me about at time when you were not able to use these pages.
      1. What happened?
      2. What got in the way?
6. Sometimes, life gets very busy. Was it ever so busy that you it was hard to do any of the activities we’ve been talking about?
   1. What happened?
   2. What did you do to help you do the activities even though it was so busy?
   3. What happened when you were not able to do the activities?
7. [If withdrew from trial] Tell me more about what was happening when you decided to stop being part of the study.
   1. What made you and your family decide to stop being part of the study?

**Part 5 – Reflecting on Trial Experiences**

1. So, we’ve talked about taking medicine, eating the powder, collecting poop in containers, and using the brochure.
   1. Which of these was the hardest to do?
   2. Which of these was the easiest to do?
2. Thinking about the four activities we talked about, what would have made them easier to do?
   1. What would have made it easier to take your medicine?
   2. What would have made it easier to eat/drink the powder?
   3. What would have made it easier to put your poop in jars?
   4. What would have made it easier to use the study booklet?
3. What would you tell other children who are about to start participating in the study?
   1. What would you want to tell them to help them get ready?

Is there anything else you’d like to share with me today?

Thank you so much for taking the time to speak with me!
